# nanoCAGE reveals 5′ UTR features that define specific modes of translation of functionally related MTOR-sensitive mRNAs

**DOI:** 10.1101/gr.197566.115

**Published:** 2016-05

**Authors:** Valentina Gandin, Laia Masvidal, Laura Hulea, Simon-Pierre Gravel, Marie Cargnello, Shannon McLaughlan, Yutian Cai, Preetika Balanathan, Masahiro Morita, Arjuna Rajakumar, Luc Furic, Michael Pollak, John A. Porco, Julie St-Pierre, Jerry Pelletier, Ola Larsson, Ivan Topisirovic

**Affiliations:** 1 Lady Davis Institute, SMBD Jewish General Hospital, Montreal, Canada H3T 1E2;; 2 Department of Oncology, McGill University, Montreal, Canada H3G 1Y6;; 3 Department of Experimental Medicine, McGill University, Montreal, Canada H3G 1Y6;; 4 Department of Biochemistry, McGill University, Montreal, Canada H3G 1Y6;; 5 Department of Oncology-Pathology, Science for Life Laboratory, Karolinska Institutet, 171 65 Solna, Sweden;; 6 Goodman Cancer Research Centre, McGill University, Montreal, Canada H3A 1A3;; 7 Cancer Program, Biomedicine Discovery Institute and Department of Anatomy and Developmental Biology, Monash University, Victoria 3800, Australia;; 8 Center for Chemical Methodology and Library Development, Boston University, Boston, Massachusetts 02215, USA

## Abstract

The diversity of MTOR-regulated mRNA translation remains unresolved. Whereas ribosome-profiling suggested that MTOR almost exclusively stimulates translation of the TOP (terminal oligopyrimidine motif) and TOP-like mRNAs, polysome-profiling indicated that MTOR also modulates translation of mRNAs without the 5′ TOP motif (non-TOP mRNAs). We demonstrate that in ribosome-profiling studies, detection of MTOR-dependent changes in non-TOP mRNA translation was obscured by low sensitivity and methodology biases. Transcription start site profiling using nano-cap analysis of gene expression (nanoCAGE) revealed that not only do many MTOR-sensitive mRNAs lack the 5′ TOP motif but that 5′ UTR features distinguish two functionally and translationally distinct subsets of MTOR-sensitive mRNAs: (1) mRNAs with short 5′ UTRs enriched for mitochondrial functions, which require EIF4E but are less EIF4A1-sensitive; and (2) long 5′ UTR mRNAs encoding proliferation- and survival-promoting proteins, which are both EIF4E- and EIF4A1-sensitive. Selective inhibition of translation of mRNAs harboring long 5′ UTRs via EIF4A1 suppression leads to sustained expression of proteins involved in respiration but concomitant loss of those protecting mitochondrial structural integrity, resulting in apoptosis. Conversely, simultaneous suppression of translation of both long and short 5′ UTR mRNAs by MTOR inhibitors results in metabolic dormancy and a predominantly cytostatic effect. Thus, 5′ UTR features define different modes of MTOR-sensitive translation of functionally distinct subsets of mRNAs, which may explain the diverse impact of MTOR and EIF4A inhibitors on neoplastic cells.

Genome-wide gene expression studies have mostly focused on measuring “steady-state” mRNA abundance that reflects alterations in transcription and mRNA stability (Piccirillo et al. 2014). Changes in steady-state mRNA abundance do not, however, completely mirror those occurring in the corresponding proteome (Gygi et al. 1999; de Sousa Abreu et al. 2009; Larsson et al. 2012; Kristensen et al. 2013; Ly et al. 2014). Although still debated (Li and Biggin 2015), these studies implicate post-transcriptional mechanisms, including mRNA translation, as key influencers over the proteome (Schwanhausser et al. 2011; Vogel and Marcotte 2012; Kristensen et al. 2013; Li et al. 2014; Jovanovic et al. 2015).

Control of translation largely occurs at the initiation step, during which the mRNA is recruited to the ribosome by the eIF4F complex (Hinnebusch 2014). This complex consists of the mRNA cap-binding protein EIF4E, a large scaffolding protein EIF4G, and DEAD box helicase EIF4A (Hinnebusch 2014). eIF4F complex assembly is regulated by the mechanistic/mammalian target of rapamycin (mTOR) complex 1 (mTORC1), which phosphorylates and inactivates 4E-binding proteins (EIF4EBP1, 2, and 3) (von Manteuffel et al. 1996; Hara et al. 1997; Burnett et al. 1998; Gingras et al. 1999, 2001). EIF4EBPs bind to EIF4E, thereby preventing EIF4E:EIF4G association (Pause et al. 1994a). Phosphorylation of EIF4EBPs leads to their dissociation from EIF4E, allowing EIF4E:EIF4G interaction and eIF4F assembly (Gingras et al. 1999, 2001). Although EIF4E is required for cap-dependent translation of all nuclear-encoded mRNAs, a subset of mRNAs characterized by long and complex 5′ UTRs that encode proliferation- (e.g., cyclins), survival- (e.g., BCL2 family members), and tumor-promoting proteins (e.g., MYC) are thought to be particularly sensitive to EIF4E (Koromilas et al. 1992; Graff et al. 2008; Roux and Topisirovic 2012; Pelletier et al. 2015).

UTR features dictate translation efficiency (Sonenberg and Hinnebusch 2009). In mammals, mRNAs with long, complex 5′ UTRs exhibit a high EIF4A helicase requirement for scanning of 43S complex toward the initiation codon (Svitkin et al. 2001). EIF4A processivity, as a single protein, is low (Pause et al. 1994b). EIF4A:EIF4G interaction dramatically increases EIF4A processivity (Garcia-Garcia et al. 2015). EIF4E recruits EIF4A to the eIF4F complex, thereby bolstering EIF4A activity (Feoktistova et al. 2013). Recent ribosome-profiling studies indicate that the mTORC1/EIF4EBP/EIF4E pathway almost exclusively regulates translation of mRNAs harboring 5′ terminal oligopyrimidine (5′ TOP) and related motifs (Hsieh et al. 2012; Thoreen et al. 2012). The 5′ TOP motif consists of a C immediately after the mRNA cap, followed by 4–15 pyrimidines (Meyuhas and Kahan 2015). TOP mRNAs encode translational machinery components, including ribosomal proteins and elongation factors (Meyuhas and Kahan 2015).

The generality of the conclusions that EIF4EBPs are major regulators of TOP mRNA translation has been questioned, because under a number of conditions, translation of TOP mRNAs, albeit MTOR-dependent, is EIF4EBP-independent (Miloslavski et al. 2014). Consistent with this view, the effects of serum deprivation on TOP mRNA translation also appeared to be EIF4E-independent (Shama et al. 1995). In turn, LARP1 and TIA1/TIAL1 (also known as TIAR) were reported to mediate effects of mTORC1 on TOP mRNAs (Damgaard and Lykke-Andersen 2011; Tcherkezian et al. 2014; Fonseca et al. 2015). Polysome-profiling showed that the mTORC1/EIF4EBP axis also regulates translation of non-TOP mRNAs, including those encoding mitochondrial and survival- and proliferation-promoting proteins (Larsson et al. 2012; Morita et al. 2013). In fact, it appears that ribosome- and polysome-profiling studies provided strikingly different sets of MTOR-sensitive mRNAs.

Elucidating the precise mechanisms that control mRNA translation also requires accurate knowledge of transcription start sites (TSSs). Despite substantial efforts to define TSSs using CAGE/nanoCAGE (The FANTOM Consortium and the RIKEN PMI and CLST [DGT] 2014) or TSS-seq (Suzuki and Sugano 2003; Suzuki et al. 2015), there are no resources that provide TSS information for the most commonly used model cell lines. Databases such as RefSeq or UCSC are thus routinely used to infer the link between 5′ UTR features and translation. These databases are thought to contain numerous errors, and for a more accurate understanding of the relationship between the 5′ UTR features and translational control, both TSSs and translation efficiency should be determined in the same cell.

## Results

### Simulating the impact of differences in polysome association on performance of ribosome- and polysome-profiling

**Figure 1.**
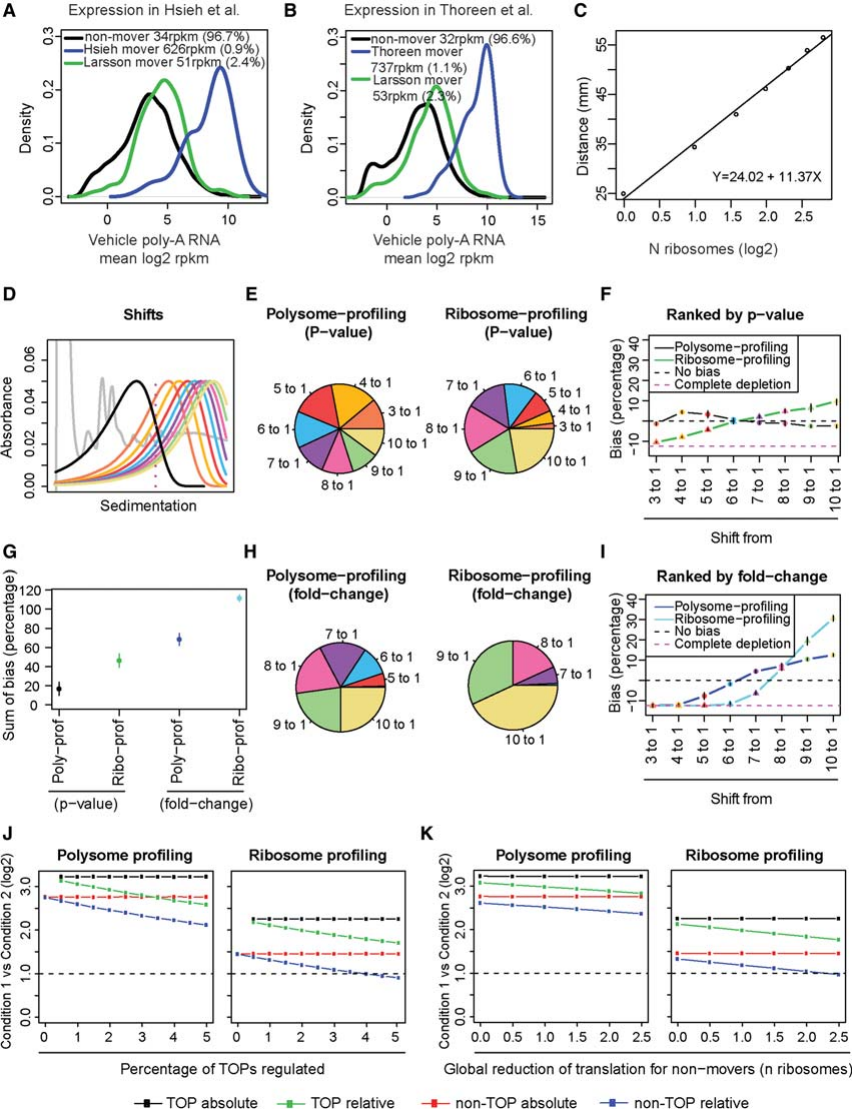
Biases in polysome- and ribosome-profiling studies of translation efficiency. (*A*,*B*) Mean expression levels and densities according to data from poly(A)^+^ RNA for mRNAs identified as differentially translated by ribosome-profiling (Hsieh et al. 2012; Thoreen et al. 2012), which include ∼60%–70% TOP mRNAs; polysome-profiling (Larsson et al. 2012), which includes ∼10% TOP mRNAs; and nonmovers. Percentage of mRNAs and mean reads per kilobase per million mapped reads (RPKM) per subset is indicated. (*C*) Calculated relationship between number of bound ribosomes and sedimentation in a 5%–50% sucrose gradient. (*D*) Simulated distributions of ribosome association under a condition when MTOR is inhibited (black) and a range of ribosome association under the control condition. (*E*,*H*) Proportions of differentially translated mRNAs identified by polysome- or ribosome-profiling simulations from analyzed shifts in polysome association. Genes were ranked by *P*-value (*E*) or fold change (*H*). (*F*,*I*) Observed bias (i.e., obtained percentage of genes from each shift compared to expected percentage) across all shifts. (*G*) Sums of bias across all shifts for each technology and analysis approach. (*F*,*G*,*I*) Means and standard deviations from four replicated simulations are shown. (*J*,*K*) Observed absolute or relative fold changes using polysome- or ribosome-profiling for TOP and non-TOP mRNAs under conditions when an increasing proportion of TOP mRNAs change their translation (*J*) or when global translation is affected (*K*).

We compared ribosome- and polysome-profiling for their ability to identify mRNAs undergoing different shifts in translational efficiency. In polysome-profiling, translation efficiency is directly determined by separating efficiently (commonly defined as mRNAs associated with more than three ribosomes) and not efficiently translated mRNAs (defined as mRNAs associated with three or less ribosomes) (Gandin et al. 2014). In contrast, with ribosome-profiling translational efficiency is inferred based on the number of RNA sequencing reads corresponding to ribosome-protected fragments (Gandin et al. 2014; Ingolia 2014; King and Gerber 2014). TOP mRNAs are highly expressed (Fig. 1A,B), engage a considerable proportion of cellular ribosomes when MTOR is active (Levy et al. 1991; Meyuhas and Kahan 2015; Miloslavski et al. 2014), and show large shifts in translational efficiency upon MTOR inhibition, as compared to mRNAs that do not contain a 5′ TOP motif (non-TOP mRNAs) (Supplemental Fig. 1; Levy et al. 1991; Miloslavski et al. 2014).

We therefore determined performance of ribosome- vs. polysome-profiling in identifying differentially translated mRNAs as a function of the size of shifts in translational efficiency using a simulation approach. Suitable parameters for simulation were obtained by comparisons of modeled normal distributions of ribosome association converted to sedimentation distances (Fig. 1C) to experimentally determined ribosome associations in insulin- vs. insulin + torin1-treated MCF7 cells (Supplemental Fig. 2A–C). Using these parameters, we then modeled a range of shifts in control vs. MTOR inhibition conditions (Fig. 1D) and estimated the mean number of ribosomes per mRNA (i.e., ribosome-profiling) (Ingolia et al. 2009; Ingolia 2014) or the proportion of the mRNAs that are associated with more than three ribosomes (i.e., polysome-profiling) (Larsson et al. 2012). We calculated fold-change differences and their *p*-values (control vs. MTOR inhibition) for each shift, for both polysome- and ribosome-profiling simulations. Because equal proportions of all shifts were represented in the simulation, the proportion of each shift among the smallest *P*-values or largest fold changes is expected to be the same. Using *P*-value-based ranking, polysome-profiling appeared not to be affected by the size of the shift in polysome association, while ribosome-profiling was biased toward identifying mRNAs that exhibit larger shifts (Fig. 1E–G). Similar bias was observed for polysome-profiling when fold-change analysis was employed, but polysome-profiling still outperformed ribosome-profiling (Fig. 1H,I). These analyses were stable inasmuch as modifying the simulation parameters did not alter the outcome (Supplemental Fig. 3).

Using the same approach, we compared performance of ribosome- vs. polysome-profiling in identifying changes of non-TOP vs. TOP mRNA translation as a function of MTOR activity. Parameters were determined from experimentally tested TOP and non-TOP mRNAs (Supplemental Figs. 1, 2). Shifts to sub-80S fractions cannot be modeled using normal distributions, and the mean of the distribution under MTOR inhibition was therefore set to one ribosome. While polysome-profiling identified expected equal proportions of TOP and non-TOP mRNAs among genes with the lowest *P*-values, ribosome-profiling again favored identification of TOP mRNAs as differentially translated (Supplemental Fig. 4A). Analysis based on fold changes showed bias for identification of TOP mRNAs over non-TOP mRNAs as differentially translated for both polysome- and ribosome-profiling, but the bias toward identifying TOP mRNAs as differentially translated was still less pronounced for polysome-profiling (Supplemental Fig. 4B,C). Thus, ribosome-profiling shows a bias which favors identification of mRNAs that exhibit larger shifts in polysomes as translationally regulated, which is the case for TOP mRNAs after modulation of MTOR activity.

Gene expression is commonly quantified by relative rather than absolute measurements. Accordingly, changes in each specific mRNA translation are measured in relation to the mean effect on translation of all mRNAs. To account for this, we performed simulations using relative translation efficacies and included invariant mRNAs (i.e., those mRNAs that did not appear to change significantly their translation in response to MTOR inhibition in both ribosome- [Hsieh et al. 2012] and polysome-profiling studies [Larsson et al. 2012], hereafter referred to as “nonmovers”). Simulation parameters for ribosome association for nonmovers were obtained by fitting a normal distribution to the polysome-profile (Supplemental Fig. 2A). Changes in translation were then simulated as described above for TOP and non-TOP mRNAs while including nonmovers. We assessed how increasing the proportion of TOP mRNAs that changed their translation impacts predicted fold changes for TOP- and non-TOP mRNAs. This was done before and after normalization by global centering (which is equivalent to an absolute and relative assessment of effects on translation, respectively). For relative measurements, when an increased proportion of TOP mRNAs were differentially translated, there was an underestimation of the fold changes for both TOP and non-TOP mRNAs (Fig. 1J). This effect was more pronounced for ribosome-profiling, e.g., if ∼3.5% of all mRNAs are TOP mRNAs and they changed translation, non-TOP mRNAs will show a less than twofold difference, which is a commonly used threshold.

As MTOR inhibition also involves a global down-regulation of protein synthesis (Roux and Topisirovic 2012), we assessed how this would impact on TOP and non-TOP mRNA translation. For this simulation we used the parameters identified for abundance and proportions of TOP mRNAs, non-TOP mRNAs, and nonmovers (Fig. 1A), and gradually introduced a reduction in translation for nonmover mRNAs to simulate reduction in global protein synthesis caused by MTOR inhibition (Fig. 1K). In this case, as above, non-TOP mRNAs exhibited a less than twofold change when ribosome-profiling was simulated. Altogether, methodological features of ribosome-profiling introduce a bias that obscures identification of changes in non-TOP mRNA translation. This is because non-TOP mRNAs show smaller shifts and lower abundance and are therefore more sensitive to global changes in protein synthesis as compared to TOP mRNAs. This is likely a key factor underpinning the discrepancies between the repertoires of MTOR-regulated mRNAs observed in ribosome- vs. polysome-profiling studies.

### Low sensitivity limits the catalog of MTOR-sensitive mRNAs identified by ribosome-profiling

Optimal RNA-seq depth under control conditions (i.e., vehicle- or mock-treated cells) is a prerequisite to identify a reduction in translation induced by a treatment (Larsson and Nadon 2013). We observed that <2000 genes consistently had >256 ribosome-profiling counts in the control condition (Supplemental Fig. 5A) across the two replicates reported in Hsieh et al. (2012). Indeed, low read counts strongly influenced the signal to variance relationship, and the variance strongly increased at <64 reads (Supplemental Figs. 5B, 6A). Using edgeR analysis (see below), we observed that the vast majority of MTOR-sensitive mRNAs did not shift below the <64-read threshold upon MTOR inhibition (Supplemental Fig. 5B). This suggests that only abundant mRNAs (e.g., TOP mRNAs) (Fig. 1A,B) would be identified as being translationally suppressed by MTOR inhibitors (MTOR-i) in this study (Hsieh et al. 2012). Indeed, <10% of non-TOP mRNAs, identified as being MTOR-sensitive in a polysome-profiling study (Larsson et al. 2012), consistently obtained >256 ribosome-profiling counts in the control condition in Hsieh et al. (2012). Alternative algorithms corroborated these findings (Supplemental Fig. 6B). A second ribosome-profiling data set investigating MTOR-sensitive translation (Thoreen et al. 2012) also suffered from this type of low sensitivity and could not identify non-TOP mRNAs as being differentially translated (Supplemental Figs. 5C,D, 7A,B). Thus, despite the differences in cell lines and conditions that were used in respective studies (Hsieh et al. 2012; Larsson et al. 2012; Thoreen et al. 2012), discrepancies in the observed effects of MTOR-i on the translatome between ribosome- and polysome-profiling studies appear to stem from technical biases and the low sensitivity in ribosome-profiling studies, and our simulation analysis indicates that, in addition to TOP mRNAs, MTOR regulates translation of non-TOP mRNAs.

**Figure 2.**
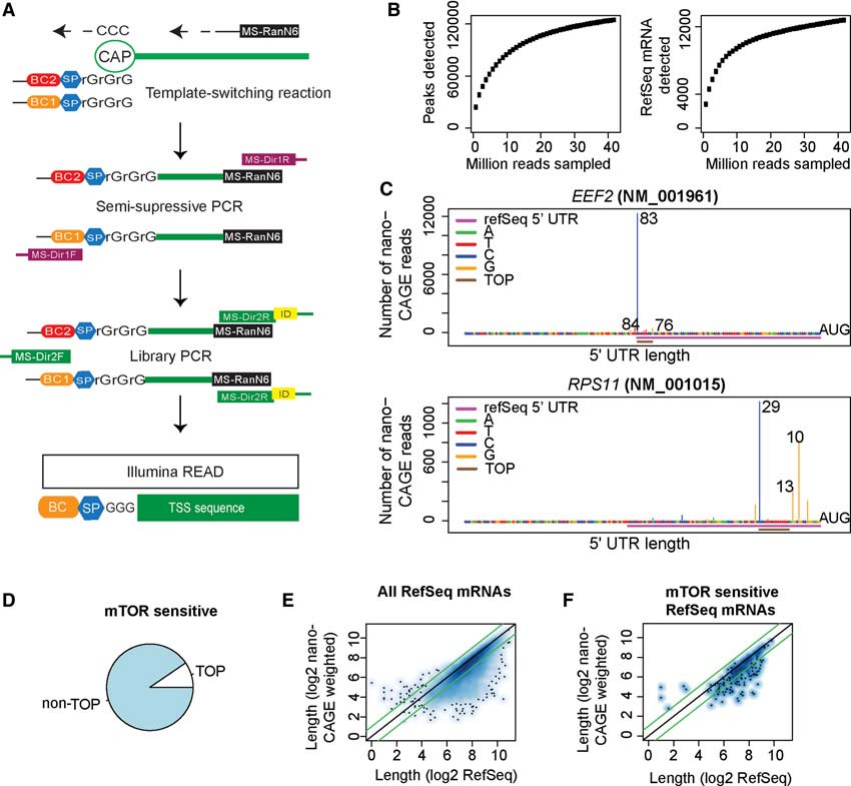
Transcription start site analysis using nanoCAGE confirms that MTOR regulates translation of non-TOP mRNAs. (*A*) The nanoCAGE procedure to identify transcription start sites (TSS). (BC, barcode; SP, spacer; MS-RanN6, random hexamer; MS-Dir1R/F, primers for semisuppressive PCR; MS-Dir2R/F, primers for adaptor PCR). (*B*) A comparison of the number of identified peaks (more than five reads; *left*) and RefSeq mRNAs (>50 reads; *right*) under increasing number of sampled nanoCAGE sequencing reads. (*C*) TSS revealed by nanoCAGE in two TOP mRNAs. The UTR lengths suggested by the three major TSS peaks are indicated together with the 5′ UTR region according to RefSeq and the position of the 5′ TOP motif. (*D*) The proportion of MTOR-sensitive (by polysome-profiling) mRNAs with TOP elements according to nanoCAGE. (*E*,*F*) A comparison between 5′ UTR lengths according to RefSeq and nanoCAGE mean lengths for all RefSeq mRNAs detected (*E*) and MTOR-sensitive genes (*F*). Green lines indicate a twofold difference in 5′ UTR length.

### nanoCAGE identifies subsets of MTOR-sensitive non-TOP mRNAs that differ in 5′ UTR features and functions

The above analysis indicates that other 5′ UTR features beyond 5′ TOP motifs must render mRNA translation MTOR-sensitive. We employed nanoCAGE sequencing (Salimullah et al. 2011) to map TSSs and 5′ UTRs to investigate this hypothesis (Fig. 2A). When we reached a sequencing “plateau,” where few additional genes or TSSs were identified upon additional sequencing (Fig. 2B), we had obtained 5′ UTR sequence information for ∼12,000 RefSeq transcripts, corresponding to ∼6500 genes in MCF7 cells maintained under the control, mock-treatment condition used when examining MTOR-sensitive translation by polysome-profiling (Supplemental Table S1; Larsson et al. 2012). Examination of a selection of TOP mRNAs showed that nanoCAGE accurately captured TSSs giving rise to mRNAs with TOP motifs also when these were located downstream from the TSS suggested by RefSeq (Fig. 2C). TSSs identified by nanoCAGE confirmed that most of the MTOR-regulated mRNAs identified by polysome-profiling (PP242-sensitive) (Larsson et al. 2012) do not contain a 5′ TOP motif (∼90%) (Fig. 2D).

**Figure 3.**
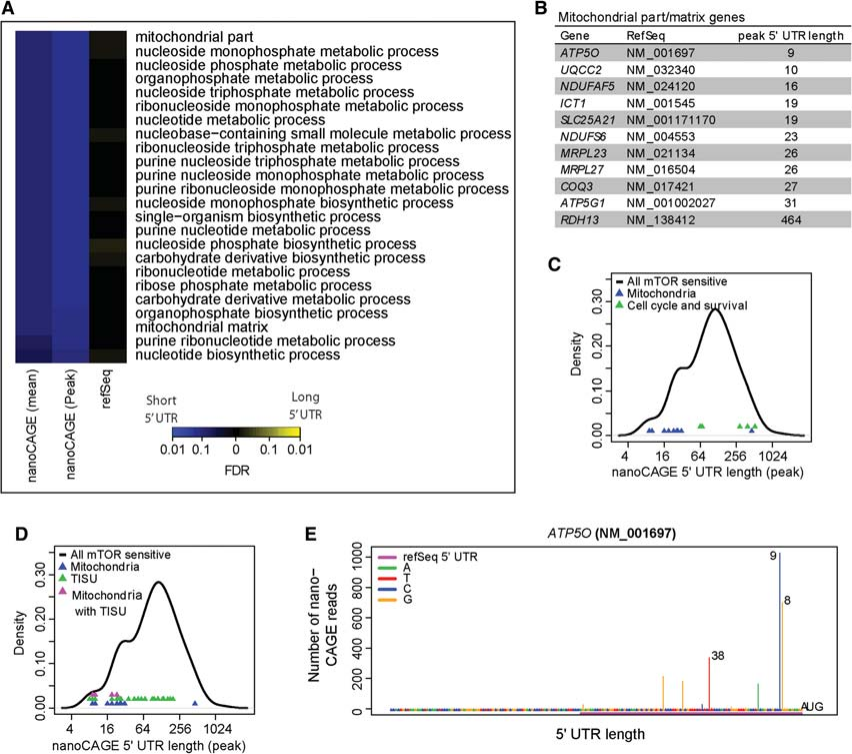
Mitochondria-related transcripts are enriched among MTOR-sensitive mRNAs with extremely short 5′ UTRs. (*A*) Enrichment of biological processes in MTOR-sensitive mRNAs (from polysome-profiling) depending on nanoCAGE or RefSeq 5′ UTR lengths. Colors represent false discovery rates for enrichments among short (blue) or long (yellow) 5′ UTRs. Both mean and largest peak 5′ UTR lengths from nanoCAGE were used as input. (*B*) A table of genes from the mitochondria-related functions (identified in *A*) and their peak 5′ UTR lengths as determined by nanoCAGE. (*C*) A comparison of 5′ UTR lengths of MTOR-sensitive mitochondrial function or cell cycle/survival related genes. (*D*) Comparison of 5′ UTR lengths and the presence of a TISU element in the indicated subsets of MTOR-sensitive mRNAs. (*E*) ATP5O is a subunit of complex V of the electron transport chain (ATP synthase) and is encoded by mRNA with a putative TISU motif and an extremely short 5′ UTR (plot as in Fig. 2C).

Determination of 5′ UTR lengths using the most prevalent peak length (i.e., the length suggested by the position with the most RNA-seq reads) or the weighted mean length (allowing all RNA-seq reads to contribute to a mean) revealed that ∼30% 5′ UTRs identified by nanoCAGE were half the length or shorter than those described in the RefSeq database (Fig. 2E,F; Supplemental Fig. 8A,B). Among the MTOR-sensitive mRNAs identified by polysome-profiling, the TSSs determined by nanoCAGE, but not RefSeq, indicated a set of transcripts with extremely short 5′ UTRs (<30 nucleotides [nt]). This set was enriched in mRNAs encoding proteins with mitochondrial functions (e.g., ATP5O, ATP5G1) (Fig. 3A,B), with one exception, RDH13, which had a long 5′ UTR. These were in stark contrast to cell cycle- and survival-promoting mRNAs with long 5′ UTRs (e.g., *CCND3, ODC1, MCL1, BIRC5* [survival] and *MYC*) (Fig. 3C). mRNAs with short 5′ UTRs, encoding proteins with mitochondrial function, were enriched (4.7-fold enrichment, *P* = 0.03) for translation initiator of short 5′ UTR (TISU) elements (Fig. 3D,E; Elfakess et al. 2011). Thus, in addition to TOP mRNAs, MTOR-sensitive mRNAs include non-TOP mRNAs with long 5′ UTRs, which encode proliferation- and survival-promoting proteins, and a subset of mRNAs with short (<30 nt) 5′ UTRs that were enriched with TISU elements (Fig. 3A–E).

### Expression of proteins encoded by a subset of MTOR-sensitive mRNAs harboring short 5′ UTRs requires EIF4E but is largely unaffected by EIF4A1

**Figure 4.**
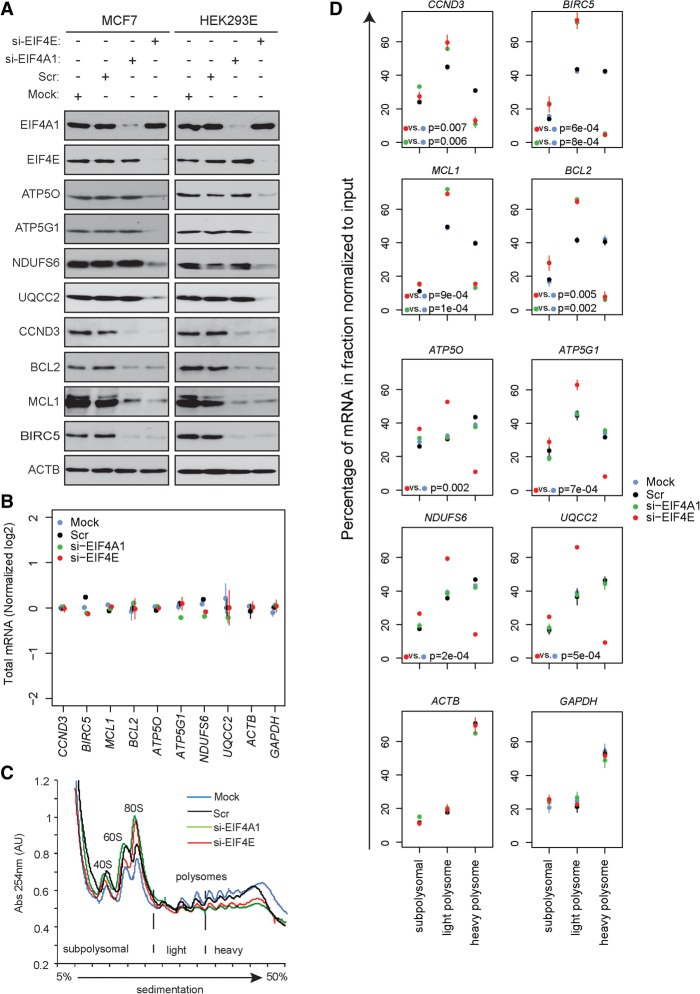
Expression of proteins encoded by mRNAs with long 5′ UTRs, but not those harboring short 5′ UTRs/TISU elements, is dependent on EIF4A1. (*A*) HEK293E and MCF7 cells were mock-transfected or transfected with siRNA targeting *EIF4A1* (si-EIF4A1), *EIF4E* (si-EIF4E), or scrambled control siRNA (Scr). Levels of indicated proteins were determined 48 h post-transfection by Western blotting. ACTB served as a loading control. Experiments were carried out in independent triplicate and quantified using densitometry (Supplemental Fig. 9). (*B*) Total levels of indicated mRNAs isolated from cells described in *A* were determined by RT-qPCR. Data were log_2_ transformed, and values obtained for indicated mRNAs were normalized to those obtained for *ACTB* and to the mean expression per gene. (*C*) Subpolysomal, light polysome, and heavy polysome fractions were obtained from cytosolic extracts from cells described in *A* by ultracentrifugation using 5%–50% sucrose gradients. During fractionation, UV absorbance at 254 nm (Abs 254 nm) was continuously monitored to obtain absorbance tracings. Positions of 40S and 60S ribosomal subunits, monosome (80S), and polysomes are indicated. (*D*) Amount of indicated mRNAs in subpolysomal, light polysome, and heavy polysome fractions isolated from cells described in *C* were determined by RT-qPCR. Experiments in panels *B* and *D* were carried out in independent duplicates, each consisting of a triplicate. Data are expressed as a percentage of a given mRNA in each fraction. Bars represent SD values. *P*-values from one-way ANOVAs for heavy polysomes are indicated.

TISU elements render mRNA translation sensitive to MTOR but not to EIF4A inhibition (Elfakess et al. 2011). We therefore depleted EIF4E or EIF4A1 in MCF7 and HEK293E cells and monitored expression of proteins encoded by MTOR-sensitive mRNAs with either long (*CCND3*, *MCL1*, *BIRC5*, and *BCL2*) or short (*ATP5O*, *ATP5G1*, *NDUFS6*, and *UQCC2*) 5′ UTRs. Whereas EIF4E depletion decreased expression of both subsets of proteins, reduction in EIF4A1 selectively affected proteins encoded by long 5′ UTR mRNAs (Fig. 4A; Supplemental Fig. 10A). We replicated these results using the active-site MTOR-i torin1 and two chemically distinct EIF4A inhibitors (EIF4A-i), hippuristanol and silvestrol (Bordeleau et al. 2005, 2006, 2008). As expected, treatment with torin1 abolished mTORC1 signaling (Thoreen et al. 2009; Galicia-Vazquez et al. 2012), whereas hippuristanol slightly induced mTORC1 activity as illustrated by changes in EIF4EBP1 and RPS6 phosphorylation (Supplemental Fig. 10B). Torin1 increased EIF4E:EIF4EBP1 and reduced EIF4E:EIF4G1 binding. In contrast, EIF4E:EIF4G1 association was unaffected by hippuristanol (Supplemental Fig. 10C). Thus, as noted following depletion of EIF4E, torin1 reduced expression of proteins encoded by both short and long 5′ UTR MTOR-sensitive mRNAs, whereas EIF4A-i selectively down-regulated proteins encoded by long 5′ UTR mRNAs (Supplemental Fig. 10B). These effects appeared to be mediated by the EIF4A1 isoform, as EIF4A2 depletion failed to decrease expression of CCND3, which is encoded by a prototypical long 5′ UTR mRNA (Supplemental Fig. 10D). As observed previously, EIF4A1 depletion led to an increase in EIF4A2 levels (Supplemental Fig. 10D; Galicia-Vazquez et al. 2012). Collectively, these results demonstrate that EIF4A1 regulates expression of proteins encoded by a subset of MTOR-sensitive mRNAs harboring long, but not short, 5′ UTRs.

### EIF4A1 affects translation of MTOR-sensitive mRNAs with long, but not short, 5′ UTRs

Depletion of EIF4E and EIF4A1 did not affect steady-state levels of mRNAs with either long (*CCND3*, *MCL1*, *BIRC5*, and *BCL2*) or short (*ATP5O*, *ATP5G1*, *NDUFS6*, and *UQCC2*) 5′ UTRs (Fig. 4B). Under these conditions, the effects of EIF4E or EIF4A1 depletion on global mRNA translation were comparable (Fig. 4C). Whereas EIF4E down-regulation decreased translation of both long and short 5′ UTR mRNAs, EIF4A1 depletion decreased translation of mRNAs with long 5′ UTRs but only marginally altered translation of mRNAs with short 5′ UTRs (Fig. 4D; Supplemental Fig. 11A). Neither EIF4A1 nor EIF4E depletion altered distribution of MTOR-insensitive mRNAs, e.g., *ACTB* (beta actin) and *GAPDH* mRNAs (Fig. 4D; Supplemental Fig. 11A). Parallel results were obtained using torin1 and hippuristanol (Supplemental Fig. 11B,C).

**Figure 5.**
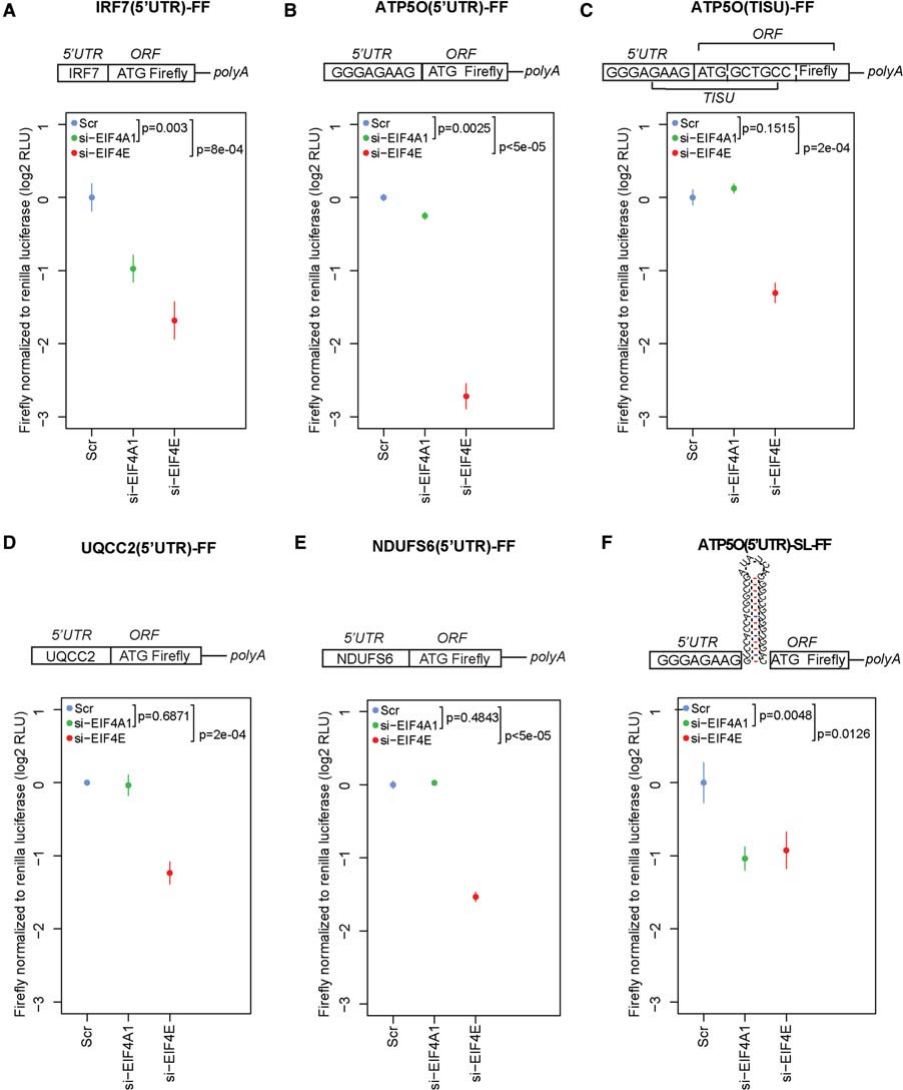
Translation of reporter mRNAs harboring short 5′ UTRs is sensitive to EIF4E, but not EIF4A1. HEK293E cells were transfected with a control (Scr), *EIF4E* (si-EIF4E), or *EIF4A1* (si-EIF4A1) siRNAs and then transfected with firefly (FF) reporters harboring *IRF7* 5′ UTR (IRF7[5′UTR]-FF) (*A*), *ATP5O* 5′ UTR with a proximal portion of TISU element before the initiation codon (ATP5O[5′UTR]-FF) (*B*), *ATP5O* 5′ UTR with a full TISU element (ATP5O[TISU]-FF) (*C*), *UQCC2* 5′ UTR (UQCC2[5′UTR]-FF) (*D*), *NDUFS6* 5′ UTR (NDUFS6[5′UTR]-FF) (*E*), or *ATP5O* 5′ UTR followed by a stem–loop structure (ATP5O[5′UTR]-SL-FF) (*F*). As a control, cells were cotransfected with a *Renilla* reporter. Luminescence was monitored 48 h post-transfection. Data for firefly luminescence normalized to *Renilla* luminescence were log_2_ transformed and normalized per replicate and to the mean of Scr (see Supplemental Fig. 14). Each experiment was performed in independent triplicates, each consisting of three replicates, and data are shown as mean ± SD. RLU, relative light units. Schematics of the FF constructs are provided in the *above* histograms, and the positions of TISU and ORF of FF luciferase are indicated. *P*-values from one-way ANOVAs are shown.

To further identify 5′ UTR features that render MTOR-sensitive mRNAs largely insensitive to changes in EIF4A1 activity, HEK293E cells were cotransfected with a *Renilla* (control) and a variety of firefly (FF) luciferase reporters. FF reporters included *IRF7* 5′ UTR (IRF7[5′UTR]-FF), which is long and translated in an EIF4E-dependent manner (Fig. 5A; Colina et al. 2008), *ATP5O* 5′ UTR (ATP5O[5′UTR]-FF), which contains a 4-nt portion of the TISU element upstream of the initiation codon (Fig. 5B), and *ATP5O* with a complete TISU element, including a 5-nt portion distal to the initiation codon (ATP5O[TISU]-FF) (Fig. 5C). *ATP5O*(5′UTR)-FF therefore harbors a disrupted TISU element whereby the TISU element sequence is substituted with GAGAA (the first 5 nt of the FF luciferase ORF) downstream from the initiation codon. EIF4E down-regulation strongly decreased expression of all three FF reporters. In contrast, while EIF4A1 depletion strongly repressed luciferase activity of *IRF7*-FF, it only had a moderate effect on *ATP5O*(5′UTR)-FF (1.2-fold reduction vs. 6.6-fold observed in EIF4E-depleted cells) and did not significantly affect luciferase activity driven by ATP5O(TISU)-FF (Fig. 5A–C). Similar results were obtained with torin1 and hippuristanol (Supplemental Fig. 12A–C). As TISU element integrity did not appear to exert a major influence on EIF4A1- or EIF4E-sensitive translation (Fig. 5B,C; Supplemental Fig. 12B,C), we also monitored expression of FF luciferase driven by constructs harboring the *UQCC2* 5′ UTR that does not contain a TISU and *NDUFS6* 5′ UTR, but which, similar to ATP5O(5′UTR)-FF, only contains a portion of the TISU element upstream of the initiation codon. In both cases, results paralleled those observed using the ATP5O reporters (Fig. 5D,E). Increasing secondary structure and length by adding a stable stem–loop structure (ATP5O[5′UTR]-SL-FF) (Babendure et al. 2006) rendered the reporter both EIF4E- and EIF4A1-sensitive (Fig. 5F). Importantly, under all conditions listed above, control *Renilla* luciferase expression was largely insensitive to both EIF4E and EIF4A1 (Supplemental Figs. 13, 14). These data demonstrate that translation of MTOR-sensitive mRNAs with short 5′ UTRs is strongly sensitive to EIF4E, but not EIF4A1, while there is no obligatory requirement for an intact TISU element.

### Effects of MTOR-i and EIF4A-i on translation are reflected by changes in mitochondrial functions

**Figure 6.**
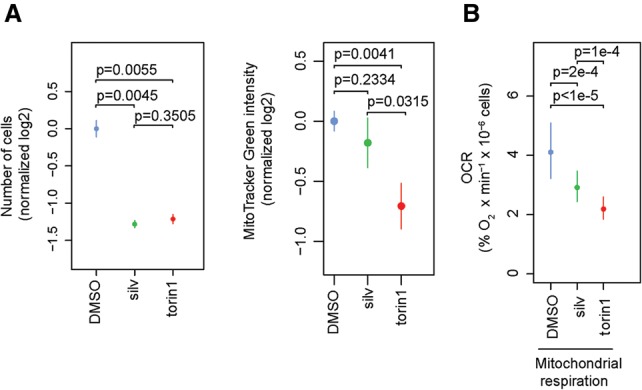
Short-term (16 h) MTOR, but not EIF4A1 inhibition, decreases mitochondrial number and respiration. (*A*) HEK293E cells were treated with torin1 (250 nM), silvestrol (silv; 25 nM), or a vehicle (DMSO) control for 16 h. Cells were counted to ensure that used concentrations of torin1 and silvestrol result in comparable inhibition of proliferation (*left* panel), and mitochondrial mass was estimated by monitoring mean fluorescence intensity of MitoTracker Green using flow cytometry (*right* panel). Data were log_2_ transformed, normalized per replicate and to the mean of DMSO, and are shown as means ± SD (*n* = 3). (*B*) HEK293E cells were treated with torin1 (250 nM), silvestrol (silv; 25 nM), or DMSO for 16 h. Mitochondrial respiration was assessed using a Clark electrode and presented as oxygen consumption rate (OCR). Data are shown as means of four independent experiments (*n* = 4) ± SD in linear scale. *P*-values from one-way ANOVAs calculated after log_2_ transformation and normalization per replicate and to DMSO are indicated.

mTORC1/EIF4EBP1/EIF4E axis bolsters mitochondrial biogenesis and ATP production by stimulating translation of nuclear-encoded mRNAs with mitochondrial functions, including respiratory chain components, e.g., *ATP5O* (Morita et al. 2013). Here, nanoCAGE identified a subset of MTOR-sensitive mRNAs encoding mitochondria-related proteins characterized by short 5′ UTRs (Fig. 3B–D), which is consistent with the findings that mRNAs encoding mitochondrial proteins frequently contain TISU elements (Sinvani et al. 2015). As translation of mRNAs with short 5′ UTRs is largely EIF4A-independent, we postulated that short-term EIF4A1 inhibition would have a lesser impact on mitochondrial number and function as compared to MTOR inhibition. To directly compare the effects of MTOR-i vs. EIF4A-i, we selected concentrations of torin1 and silvestrol that comparably suppressed HEK293E cell proliferation (∼60% of control) (Fig. 6A, left panel). After 16 h (a time period we previously found to be suitable for detecting the impact of MTOR inhibition on cell mitochondrial content and respiration [Morita et al. 2013]), torin1, but not silvestrol, reduced mitochondrial content (Fig. 6A, right panel).

In addition, although both torin1 (1.9-fold) and silvestrol (1.4-fold) reduced mitochondrial respiration, the effect of torin1 was more substantial (Fig. 6B). Similar findings were obtained using siRNA directed against *EIF4E* or *EIF4A1* (Supplemental Fig. 15A). Furthermore, whereas EIF4A1 down-regulation slightly elevated mitochondrial respiration relative to the control, mitochondrial respiration was dramatically inhibited by EIF4E depletion (Supplemental Fig. 15B). Therefore, short-term treatment with MTOR-i suppresses mitochondrial respiration more dramatically than EIF4A-i, which is consistent with the disparity of their effects on translation of mRNAs harboring short 5′ UTRs, i.e., those encoding proteins involved in mitochondrial respiration and functions.

### EIF4A-i more dramatically induces apoptosis compared to MTOR-i

**Figure 7.**
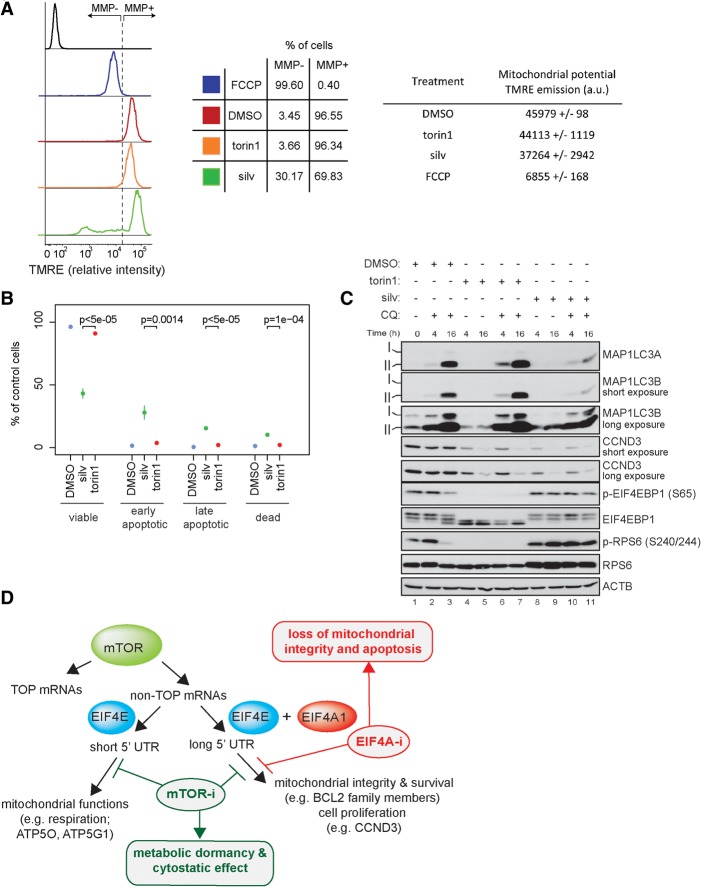
Prolonged (72 h) inhibition of EIF4A1, but not MTOR, compromises mitochondrial integrity and leads to apoptosis. (*A*) HEK293E cells were treated with torin1 (250 nM), silvestrol (silv; 25 nM), or a vehicle (DMSO) for 72 h. Mitochondrial membrane potential (MMP) was analyzed by monitoring TMRE fluorescence intensity by flow cytometry. As a control, cells were treated with FCCP (20 μM; 10 min) to induce mitochondrial membrane depolarization. (*Left* panel) The flow cytometry histogram profiles of unstained (control; black) and TMRE stained cells treated with FCCP (blue), a vehicle (DMSO; red), torin1 (orange), or silvestrol (silv; green). Cells harboring depolarized mitochondria (MMP−) were defined as those with a TMRE signal equal to or less than the TMRE signal observed in FCCP-treated cells. (*Middle* panel) Mean percentage (*n* = 3) of MMP− and MMP+ cells out of total viable cells (DAPI positive dead cells were excluded). (*Right* panel) Overall TMRE emission expressed in arbitrary units (a.u.). Results are presented as mean ± SD (*n* = 3). (*B*) HEK293E cells were treated as described in *A*, and apoptosis was measured by flow cytometry using a FITC-Annexin V/PI staining. The fractions (%) of viable (Annexin V−/PI−), early apoptotic (Annexin V+/PI−), late apoptotic (Annexin V+/PI low), and dead (Annexin V+/PI high) cells are shown relative to the total cell population. Results represent means ± SD (*n* = 3). *P*-values from one-way ANOVAs are shown. (*C*) HEK293E cells were treated with torin1 (250 nM) or silvestrol (silv; 25 nM) in the presence or absence of chloroquine (CQ, 50 µM) for 4 and 16 h. Autophagy induction was monitored by the detection of MAP1LC3A/B lipidation (MAP1LC3A-II and MAP1LC3B-II) using appropriate antibodies. The levels and phosphorylation status of indicated proteins were monitored by Western blotting. ACTB served as a loading control. (*D*) Schematic representation of the model. In addition to 5′ TOP motif (TOP mRNAs), 5′ UTR features define two distinct subsets of non-TOP mRNAs whose translation is stimulated by MTOR (mTOR; green): (1) mRNAs with long 5′ UTRs whose translation is sensitive to both EIF4E (blue) and EIF4A1 (red), which encode pro-survival- (e.g., BCL2) and proliferation-promoting (e.g., CCND3) proteins, and (2) mRNAs with short 5′ UTRs whose translation is sensitive to EIF4E (blue), but not EIF4A1 (red), which encode proteins with mitochondrial functions (e.g., ATP5O). Whereas MTOR-i (mTOR-i; green) suppresses translation of both subsets of mRNAs (1 and 2) leading to metabolic dormancy and cytostatic effect, EIF4A-i (red) selectively inhibits translation of mRNAs with long 5′ UTRs (1), leading to apoptosis.

Inhibition of the mTORC1/EIF4EBP1/EIF4E axis coincides with down-regulation of proteins implicated in mitochondrial respiratory function (e.g., ATP5O, ATP5G1, UQCC2, NDUFS6) as well as in the maintenance of the integrity of the mitochondrial outer membrane (e.g., BCL2, MCL1, and BIRC5) (Fig. 4A). In contrast, suppression of EIF4A1 activity selectively affects proteins that maintain mitochondrial outer membrane integrity (Fig. 4A). This explains why MTOR inhibitors mostly have a cytostatic effect (Morita et al. 2015) and suggests that prolonged EIF4A1 inhibition may trigger mitochondrial-mediated apoptosis by disrupting the coordinated expression of proteins with mitochondrial metabolic functions from those regulating mitochondrial structural integrity. We therefore treated HEK293E cells with torin1 or silvestrol for 72 h and measured mitochondrial membrane potential using TMRE (tetramethylrhodamine, ethyl ester), a cell-permeant dye that accumulates in active but not depolarized mitochondria (Scaduto and Grotyohann 1999). As a positive control, cells were treated with carbonyl cyanide 4-(trifluoromethoxy)phenylhydrazone (FCCP), a compound that depolarizes mitochondria (Fig. 7A). Exposure to silvestrol (72 h) markedly induced depolarization of mitochondria (Fig. 7A), and this correlated with induction of apoptosis as compared to control or torin1-treated cells (Fig. 7B). Similar results were observed in nutrient-deprived cells depleted of EIF4E or EIF4A1 (Supplemental Fig. 15C,D). These observations indicate that suppression of translation of both long and short 5′ UTR MTOR-sensitive mRNAs by torin1 yields a predominantly cytostatic outcome, whereas suppression of EIF4A1 function induces apoptosis by exclusively inhibiting translation of mRNAs with long 5′ UTRs encoding proteins that maintain mitochondrial structural integrity.

MTOR inhibition increases autophagy (Kim and Guan 2015), which eliminates depolarized mitochondria (Elmore et al. 2001; Narendra et al. 2008; Twig et al. 2008). To determine whether EIF4A1 inhibition affects autophagy, we monitored autophagic flux by treating HEK293E cells with torin1 or silvestrol in the presence of chloroquine. Chloroquine prevents autophagosome-lysosome fusion causing accumulation of the autophagic markers MAP1LC3A-II (LC3A-II) and MAP1LC3B-II (LC3B-II) (Klionsky et al. 2012). While torin1 induced autophagy, as evidenced by elevated levels of both MAP1LC3A-II and MAP1LC3B-II (Fig. 7C), silvestrol prevented accumulation of MAP1LC3A-II and MAP1LC3B-II induced by chloroquine (Fig. 7C). By inhibiting the lysosomal pH gradient, chloroquine impairs mTORC1 activation (Settembre et al. 2012), as illustrated by the decrease in the phosphorylation of EIF4EBP1 and RPS6 after 16 h (Fig. 7C). mTORC1 inhibition by chloroquine was partially reversed by silvestrol (Fig. 7C). This is consistent with induction of mTORC1 in response to EIF4A1 inhibition (Supplemental Fig. 10B; Galicia-Vazquez et al. 2012). Therefore, in addition to inducing mitochondrial depolarization by altering mRNA translation, EIF4A-i prevents induction of autophagy and elimination of depolarized mitochondria.

## Discussion

The advantage of ribosome- over polysome-profiling is that ribosome-profiling allows determination of ribosome positioning on mRNA at single-nucleotide resolution (Ingolia et al. 2009). However, there are a number of challenges regarding interpretation of ribosome-profiling (Guttman et al. 2013; Gerashchenko and Gladyshev 2014; King and Gerber 2014; Lareau et al. 2014; Pelechano et al. 2015). The relative performance of ribosome- vs. polysome-profiling to study changes in translation efficiency also remains unclear. mRNAs exhibit different translation properties, in part conferred by their 5′ UTR features (Lodish 1974; Pelletier and Sonenberg 1985; Koromilas et al. 1992; Graff et al. 2008; Sonenberg and Hinnebusch 2009). We show that ribosome-profiling studies should be interpreted with caution when simultaneously studying subclasses of mRNAs with disparate translation properties. Ribosome-profiling efficiently identifies changes in translation of highly abundant mRNAs that show large shifts in polysome association (“class A mRNAs”) but is far less sensitive in detecting less abundant mRNAs that shift less (“class B mRNAs”). TOP mRNAs, upon MTOR inhibition, shift from heavy polysomes to subpolysomal fractions (i.e., “class A mRNAs”), whereas the majority of non-TOP mRNAs transit from intermediate-heavy to light polysomes (i.e., “class B mRNAs”) (Supplemental Fig. 1). In contrast, polysome-profiling captures the translational changes in both classes of mRNAs. This may explain discrepancies between the catalogs of MTOR-regulated mRNAs between ribosome- (Hsieh et al. 2012; Thoreen et al. 2012) vs. polysome-profiling (Larsson et al. 2012) studies. Consistently, while ribosome-profiling led to the conclusion that the mTOR/EIF4EBP1/EIF4E axis exclusively controls TOP and TOP-like mRNA translation (Hsieh et al. 2012), the same group found that EIF4E predominantly affects translation of non-TOP mRNAs implicated in ROS clearance using polysome-profiling (Truitt et al. 2015).

Length, and plausibly other 5′ UTR features, distinguishes two separate subgroups of MTOR-sensitive non-TOP mRNAs that are functionally distinct. First, there are those mRNAs harboring long 5′ UTRs that encode survival- and proliferation-promoting factors and are strongly affected by EIF4E and EIF4A1. Second, there are mRNAs that harbor short 5′ UTRs/TISU elements, translated in an EIF4E- but not an EIF4A1-sensitive manner, enriched in those encoding mitochondria-related proteins. In mammals, EIF4A1 selectively stimulates translation of mRNAs that encode survival-promoting proteins (e.g., BCL2, MCL1, and BIRC5) without altering translation of mitochondria-related short 5′ UTR mRNAs (Rubio et al. 2014; Wolfe et al. 2014; Modelska et al. 2015). In yeast, Ded1 (the ortholog of DDX3X in mammals), rather than EIF4A, seems to act as a major helicase that resolves secondary 5′ UTR structures (Sen et al. 2015). Accordingly, in yeast, changes in EIF4A activity uniformly affect translation of most mRNAs, while modulation of Ded1 primarily affects those with complex 5′ UTRs (Sen et al. 2015). Several features that distinguish yeast and mammalian EIF4A may explain these distinctions. This includes differences in the apparent stability of the eIF4F complex, as EIF4E:EIF4G but not EIF4E:EIF4G:EIF4A complexes can be purified from yeast (Merrick 2015). Moreover, MTOR-regulated auxiliary factor EIF4B (Raught et al. 2004) significantly bolsters RNA-dependent ATPase activity of mammalian but not yeast EIF4A (Merrick 2015). It is also plausible that helicases thought to be required to unwind exceptionally strong 5′ UTR secondary structures (e.g., *DHX29* and *DDX3X*) (Parsyan et al. 2011) provide an additional level of diversity to MTOR-dependent translational regulation. However, the signaling mechanisms that regulate DHX29 and DDX3X function remain to be established.

Translation of TISU mRNAs is 5′ cap-dependent, resistant to glucose deprivation but not to rapamycin, and requires EIF1:EIF4G1 interaction (Sinvani et al. 2015). We demonstrate that MTOR-i or EIF4E depletion, but not inhibition of EIF4A1, suppresses translation of short 5′ UTR mRNAs (<30 nt). This suggests that EIF4E is key for translation of mRNAs containing short 5′ UTRs and TISU elements, which is postulated to occur by facilitating recruitment of EIF4G1 and which does not appear to be affected by EIF4A-i (Supplemental Fig. 10C). Moreover, MTOR and EIF4E seem to regulate translation of *ATP5O* mRNAs independently of the TISU element downstream from the initiation codon, as well as translation of *UQCC2* mRNA that comprises a short 5′ UTR without a TISU (Fig. 5B–D). This suggests that the length of the 5′ UTR, rather than the presence of a TISU, is the primary factor that determines EIF4E- versus EIF4A1-sensitivity.

mTORC1 modulates translation of TOP mRNAs via LARP1 (Tcherkezian et al. 2014; Fonseca et al. 2015), whereas synthesis of mitochondrial, proliferation- and survival-promoting proteins is controlled by the EIF4EBP/EIF4E pathway (Larsson et al. 2012; Morita et al. 2013). Notably, 5′ UTR features further separate non-TOP MTOR-sensitive mRNAs into two distinctive groups that differ in their EIF4A1-sensitivity. MTOR-i prevent translation of short and long 5′ UTR mRNAs diminishing mitochondrial functions, accompanied by metabolic quiescence and cytostasis, whereas selective suppression of translation of a subset of long but not short 5′ UTR mRNAs by EIF4A-i leads to mitochondrial depolarization and apoptosis (Fig. 7D). These findings suggest that mTORC1 deploys distinct effectors to modulate translation of a variety of functional groups of mRNAs far beyond TOP mRNAs to regulate cellular homeostasis in response to a variety of extracellular stimuli and intracellular cues.

In addition to the differential effects of MTOR-i and EIF4A-i on translation, it is likely that these compounds exert opposing metabolic effects. MTOR-i reduce mitochondrial ATP production while suppressing ATP consumption by anabolic processes and stimulating autophagy, which facilitates the replenishment of a variety of cellular building blocks (Morita et al. 2013; Albert and Hall 2014; Shimobayashi and Hall 2014). Although EIF4A-i may also decrease ATP consumption by the translational machinery, they are likely to stimulate other anabolic processes via increasing mTORC1 activity (Supplemental Fig. 10B; Galicia-Vazquez et al. 2012). Contrary to previous reports, the effects of silvestrol on tumor cell viability cannot be accounted for by modulation of p-EIF4E status (Chu et al. 2015). EIF4A-i may therefore avoid compensatory effects seen with MTOR-i, resulting in a more effective precipitation of mitochondrial dysfunction and cell death. Indeed, unlike MTOR-i, silvestrol attenuated autophagy, which is considered a major mechanism for nutrient replenishment and clearance of depolarized mitochondria (Elmore et al. 2001; Narendra et al. 2008; Twig et al. 2008).

In conclusion, important 5′ UTR mRNA variations determine MTOR-sensitivity. Current databases do not always accurately reflect the 5′ UTR features, while certain analytical biases currently limit the accuracy of ribosome-profiling techniques for monitoring changes in translational efficiency. Finally, two functionally distinct subgroups of non-TOP MTOR-sensitive mRNAs provide the molecular basis for the superior anti-neoplastic effects of EIF4A-i over MTOR-i.

## Methods

### Simulations

Distributions of ribosome associations were obtained by sampling from a normal distribution. These were then converted to mean number of ribosomes (equivalent to ribosome-profiling) or proportions of mRNAs associated with more than three ribosomes (equivalent to polysome-profiling), followed by calculation of fold changes and *P*-values that were compared across polysome- and ribosome-profiling. Detailed protocols for simulations are provided in the Supplemental Material.

### Preparation of nanoCAGE libraries and analysis of nanoCAGE data

NanoCAGE libraries were prepared and analyzed as described (Salimullah et al. 2011) but with a number of modifications that are described in detail in the Supplemental Material.

### Cell culture, inhibitors, antibodies, and RNAi

HEK293E and MCF7 cells (ATCC) were maintained in Dulbecco's modified Eagle's medium (DMEM; Wisent) and RPMI-1640 (Wisent), respectively, supplemented with 10% fetal bovine serum (Wisent), 1% penicillin/streptomycin (Wisent), and 1% L-glutamine (Wisent). Where indicated, cells were treated with 250 nM torin1 (Tocris), 1 μM hippuristanol, and 25 nM silvestrol or the same volume of vehicle (DMSO). Lists of siRNAs, shRNAs antibodies, and dilutions that were used in Western blots are provided in the Supplemental Material.

### Polysome profiles and RT-qPCR

MCF7 cells were seeded in a 15-cm Petri dish, harvested at 80% confluency, and lysed in hypotonic lysis buffer (5 mM Tris HCl, pH 7.5, 2.5 mM MgCl_2_, 1.5 mM KCl, 100 μg/mL cycloheximide, 2 mM DTT, 0.5% Triton, 0.5% sodium deoxycholate). Polysome-profiling was carried out as described (Gandin et al. 2014). RT-qPCR was performed as previously described (Miloslavski et al. 2014). Fractions were collected as described in Gandin et al. (2014), and RNA was extracted using TRIzol according to the manufacturer's instructions. RT and qPCR were performed by SuperScript III Reverse Transcriptase and Fast SYBR Green Mastermix (Invitrogen), respectively. Experiments were done at least in independent duplicates (*n* = 2) whereby every sample was analyzed in a technical triplicate. Analyses were carried out using a relative standard curve method as described in http://www3.appliedbiosystems.com/cms/groups/mcb_support/documents/generaldocuments/cms_040980.pdf. Primers are listed in the Supplemental Material.

### Statistical analysis

We performed one-way or two-way ANOVA in R (R Core Team 2014) as indicated in the figure legends.

## Data access

nanoCAGE data from this study have been submitted to the NCBI Gene Expression Omnibus (GEO; http://www.ncbi.nlm.nih.gov/geo/) under accession number GSE77033.
